# Localization of nanospheres in pheochromocytoma-like cells following exposure to high-frequency electromagnetic fields at 18 GHz

**DOI:** 10.1098/rsos.220520

**Published:** 2022-06-29

**Authors:** Palalle G. Tharushi Perera, Denver P. Linklater, Erim Kosyer, Rodney Croft, Elena P. Ivanova

**Affiliations:** ^1^ School of Science, RMIT University, PO Box 2476, Melbourne, ViC 3001, Australia; ^2^ Faculty of Science, Engineering and Technology, Swinburne University of Technology, PO Box 218, Hawthorn, ViC 3122, Australia; ^3^ School of Psychology, Illawarra Health and Medical Research Institute, University of Wollongong, Wollongong, NSW 2522, Australia

**Keywords:** high frequency, electromagnetic fields (EMFs), 18 GHz, pc 12 neuronal cells, membrane permeability, nanosphere localization

## Abstract

Exposure to high-frequency (HF) electromagnetic fields (EMFs) at 18 GHz was previously found to induce reversible cell permeabilization in eukaryotic cells; however, the fate of internalized foreign objects inside the cell remains unclear. Here, silica core–shell gold nanospheres (Au NS) of 20 ± 5 nm diameter were used to study the localization of Au NS in pheochromocytoma (PC 12) cells after exposure to HF EMFs at 18 GHz. Internalization of Au NS was confirmed using fluorescence microscopy and transmission electron microscopy. Analysis based on corresponding scanning transmission electron microscopy energy-dispersive spectroscopy revealed the presence of the Au NS free within the PC 12 cell membrane, cytoplasm, enclosed within intracellular vesicles and sequestered in vacuoles. The results obtained in this work highlight that exposure to HF EMFs could be used as an efficient technique with potential for effective delivery of drugs, genetic material, and nanomaterials into cells for the purpose of cellular manipulation or therapy.

## 1. Introduction

The use of high-frequency (HF) electromagnetic fields (EMFs) has found broad application, ranging from telecommunications to medical imaging [1]. Recent findings have revealed that microwave (MW) radiation is able to induce a variety of transformations at the molecular level, depending mainly on the amount of energy delivered [2,3]. It was previously reported that EMFs of 18 GHz induced transient cell permeabilization in a broad selection of prokaryotic organisms and eukaryotic cell lines, including Gram-negative bacteria *Branhamella catarrhalis* ATCC 23246, *Escherichia coli* ATCC 15034, Gram-positive bacteria *Bacillus subtilis* NCIMB 3610^T^, *Kocuria rosea* CIP 71.15^T^, *Planococcus maritimus* KMM 3738, *Staphylococcus aureus* CIP 65.8^T^, *Staphylococcus aureus* ATCC 25923, *Staphylococcus epidermidis* ATCC 14990^T^, *Streptomyces griseus* ATCC 23915, yeast cell *Saccharomyces cerevisiae* ATCC 287, red blood cells [4–6] and neuron-like pheochromocytoma (PC 12) cells without compromising cell viability.

Previously, we determined PC 12 cell membrane permeabilization following exposure to 18 GHz to be a transient event (90% of cells developing permeability for 9 min), where permeability was confirmed by the uptake of silica nanospheres (NS) (diameter *d* approx. 23.5 nm). Permeability was not associated with bulk thermal effects as a heat-treated control group, exposed to similar temperatures using a Peltier plate, did not exhibit NS internalization [6]. It was hypothesized that EMF radiation might induce membrane depolarization and transient cell permeability with no change to cell viability, proliferation, and cellular metabolism [6]. Given the internalization of NS, it is important to determine the fate of the NS inside PC 12 cells. The PC 12 cell line has been widely used as a model system for studying neuronal differentiation [7], neurosecretion, cell attachment, and membrane interactions [8–11]. The ability to directly manipulate neuronal cells has important potential in therapeutics and neural network studies. PC 12 cells are referred to as having neuron-like properties due to their ability to respond to nerve growth factor (NGF) by differentiating into sympathetic ganglion neurons [12]. Upon treatment with NGF, PC 12 cells cease proliferation, and initiate the process of differentiation by extending neurites while becoming electrically excitable [12].

The aim of this study was to reveal the exact localization of gold nanospheres (Au NS) in pheochromocytoma PC 12 cells after induced transient cell permeabilization by exposure to 18 GHz radiation, employing a combination of fluorescence microscopy, transmission electron microscopy (TEM), and scanning TEM energy dispersive spectroscopy (STEM-EDS). The localisation of foreign ‘objects’ after cell permeabilisation treatment by EMFs of 18 GHz has a significant impact for understanding the potential use of EMF irradiation in the field of drug delivery and gene therapy. As with many cell poration protocols, there is a preferential route of uptake to deliver drugs, genetic material, and nano-objects to specific areas of the cell. Understanding this delivery route is vital for realizing the potential of this promising phenomenon. Induced transient cell permeabilization by exposure to 18 GHz radiation is therefore a favourable alternative to conventional cell permeabilization techniques for drug and gene delivery applications due to the lack of morphological and cytotoxic effects after exposure.

## 2. Material and methods

### 2.1. PC 12 cell culture and growth conditions

The PC 12 cell line was derived from a transplantable rat pheochromocytoma of the adrenal gland [7]. The PC 12 cell line was purchased from the American Type Culture Collection (ATCC, USA) and cultured in a complete Gibco™ RPMI medium (Thermo Fisher Scientific, Australia) supplemented with 10% Gibco™ horse serum (Thermo Fisher Scientific, Australia, HS), 5% Gibco™ foetal bovine serum (Thermo Fisher Scientific, Australia, FBS) and 1% Gibco™ penicillin/streptomycin (Thermo Fisher Scientific, Australia, PS). Supplements were stored as aliquots at −20°C. Stock solutions of the PC 12 cells were prepared in a medium containing 90% FBS and 10% DMSO and stored in liquid nitrogen. The cells were maintained at 37°C with 5% CO_2_ in a 95% humidified incubator. The medium was changed every 2 days and passaged accordingly when the confluence reached 90%.

### 2.2. Electromagnetic fields exposure and sample preparation

The EMF exposures of the PC 12 cells were carried out according to the previously developed procedure, described elsewhere [9,11]. Briefly, the MW apparatus (Lambda Technologies Vari-Wave Model LT 1500) used in the study has a variable frequency range from 5 to 18 GHz. A fixed frequency of 18 GHz power of 17 W was chosen for all exposures [9,10]. The micro Petri dish (35 mm diameter, Griener Bio One, Frickenhausen, Germany) with the cell suspension was placed on the ceramic pedestal (Pacific Ceramics, Sunnyvale, CA, USA, *ε*′ = 160, loss tangent less than 10^−3^) on the hotspot free location, identified by electric field modelling using CST Microwave Studio 3D Electromagnetic Simulation Software (CST MWS) (CST of America, Framingham, MA, USA) and experimental temperature measurements [13]. The modelling estimates that with an electric field strength and absorbed power of 78.4 V m^−1^ and 1.83e4 W m^−3^, respectively, the magnetic field strength would be 233 A/m^2^ over the sample. There is a displacement of 50 cm from the tube to the Petri dish, which results in an estimate of 466 A m^−1^.

The cell density of PC 12 used for EMF exposure was adjusted to 6 × 10^4^ cells ml^−1^ in phosphate-buffered saline (PBS) using a haemocytometer (Paul Marienfeld GmbH & Co. KG Lauda-Konigshofen, Germany). The PC 12 cell suspensions were exposed to EMF for a duration of 30 s with three cycles of exposure (90 s of total exposure) with 2 min cooling time in between each cycle. The temperature was maintained below 37°C [6]. The temperature rise in the cell suspension was monitored using a Luxtron Fiber Optic Temperature Unit (LFOTU) (LumaSense Technologies, Santa Clara, CA, USA). After the MW exposure, the sample chamber was cooled to 25°C for 2 min by inserting ice packs into the chamber. PC 12 cells grown in full serum medium were used as the non-exposed control group.

### 2.3. Cellular uptake of silica nanospheres

Fluorescent silica NS with a diameter of 23.5 ± 0.2 nm (FITC) (Corpuscular Inc, Cold Spring, NY, USA) were used to study the permeability of PC 12 cells. The membrane phospholipids were stained using red-fluorescent carbocyanine DIL (1,1′-dioctadecyl-3,3,3′,3′- tetramethylindocarbocyanine perchlorate) dye (ThermoFisher Scientific, Australia). Immediately following the EMF exposure, the NS were added to the cell suspension at a concentration of 10 µg ml^−1^. After 5 min of incubation, the samples were washed twice using PBS and centrifuged at 1300 rpm for 5 min at 25°C. The same procedure was repeated for the non-exposed controls. A 150 µl aliquot of the sample was visualized using a Fluoview FV10i-W inverted microscope (Olympus, Tokyo, Japan). Confocal laser scanning images were used to quantify NS uptake by counting the number of cells emitting the green fluorescence (FITC) in comparison to the cells that were not permeabilized. Ten different fields representative of the entire sample were analysed.

### 2.4. Localization of silica core–shell gold nanospheres

Silica core–shell Au NS with a diameter of 20 nm ± 5 nm (nanoComposix, San Diego, CA) were used to study the uptake and localization of Au NS by PC 12 cells. Immediately following the EMF exposure, the Au NS were added to the cell suspension at a concentration of 10 µg ml^−1^. After 5 min of incubation, the samples were washed twice using PBS and centrifuged at 1300 rpm for 5 min at 25°C. The same procedure was repeated for the non-exposed controls.

### 2.5. Transmission electron microscopy

After 5 min of incubation in the presence of NS following EMF exposure, cell suspensions were pelleted by centrifugation at 1300 rpm for 5 min at 25°C. The cells were then washed twice with PBS (10 mM, pH 7.4) to remove any unbound NS. The cell pellet was conditioned with 0.1 M sodium cacodylate buffer (pH 7.4). The cell pellet was then re-suspended in primary fixative of 4% paraformaldehyde and 2.5% glutaraldehyde in 0.1 M sodium cacodylate buffer overnight at 4°C and washed thrice in sodium cacodylate buffer for 10 min each. The cells were post-fixed in 1% osmium tetroxide and 1.5% potassium ferrocyanide for 1 h, followed by three washes in distilled water for 10 min each. The cells were dehydrated using a graded series of ice-cold ethanol (50%, 70% and 90%) for 15 min each. The cells were further dehydrated by passing through 100% ethanol twice followed by 100% acetone twice for 30 min each. The cells were further infiltrated with 1 : 1 ratio of acetone: Spurr's resin mixture for overnight. Afterwards, the cells were completely exchanged in 100% Spurr's resin twice for 3 h each time, under vacuum. The resin samples were polymerized at 70°C for 48 h. The final block was trimmed, and then cut into ultrathin sections (90 nm thickness) using a Leica Ultracut Ultramicrotome (Leica Microsystems, Wetzlar, Germany) with a diamond knife (Diatome, Pennsylvania, USA). Sections were placed onto 200 mesh copper grids and examined using a JEM 1010 instrument (JEOL). Approximately 40 TEM images were observed for analysis.

### 2.6. Scanning transmission electron microscopy-energy-dispersive spectroscopy

STEM-EDS was used for the elemental analysis of the Au NS in the ultrathin sections prepared as described above. STEM-EDS analysis was performed using a TEM Jeol 2100F (FEG, 200 kV) equipped with a high-angle annular dark-field detector (scanning TEM mode, STEM) and EDS.

### 2.7. Cellular morphology

The scanning electron microscope FeSEM SUPRA 40VP (Carl Zeiss, Jena, Germany) with a primary beam energy of 3 kV was used. A 100 µl aliquot of cells in PBS was placed on a glass coverslip (ProSciTech, Kirwan, Australia) in duplicate. The glass coverslips were then washed with nanopure H_2_O (resistivity of 18.2 MW cm^−1^) and dried with 99.99% purity nitrogen gas. The PC 12 cells exposed to EMF of 18 GHz were fixed in a mixture of 2.0% paraformaldehyde and 2.5% glutaraldehyde for 30 min. The cells were then dehydrated by passing through a graded ethanol series (20%, 40%, 60%, 80% and 100%) for 15 min. Before imaging, the fixed cells were subjected to gold sputtering (7 nm thick) using a NeoCoater MP-19020NCTR (JEOL, Tokyo, Japan). The same procedure was applied to controls, non-exposed PC 12 cells.

### 2.8. Cell viability

The viability of PC 12 cells was determined using the LIVE/DEAD Viability/Cytotoxicity Kit (Invitrogen) according to the manufacturer protocol. Briefly, the LIVE/DEAD Viability/Cytotoxicity Kit consists of Calcein Am and Ethidium homodimer-1 (EthD-1). Live cells were distinguished by fluorescence expression of calcein. Non-fluorescent, cell permeable calcein AM is converted to green fluorescing calcein due to intracellular esterase activity (indicative of live cells). EthD-1 is a red-fluorescing non-membrane permeable nucleic acid dye that only enters cells with damaged membranes, thereby producing a bright red fluorescence in dead cells.

The viability of the EMF-exposed cells and the controls was monitored immediately after the treatment by confocal laser scanning microscopy (CLSM) and confirmed by three independent technical replicates. Approximately 10 fields of view were analysed per sample type.

### 2.9. PC 12 cell differentiation

PC 12 cells at a density of 10^5^ cells per mL in Gibco™ 1640 Roswell Park Memorial Institute (RPMI) medium (10% HS, 5% FBS and 1% PS) were seeded onto a 10 µg mL^−1^ collagen-coated 12-well polystyrene tissue culture plate according to the manufacturer's recommended procedure. One day after plating, the full serum culture medium was replaced with a low serum medium supplemented with 50 ng mL^−1^ NGF (mouse recombinant NGF 7S, Sigma-Aldrich, Sydney, Australia). The culture medium was partially refreshed every 2 days.

### 2.10. Assessment of neurite outgrowth

A phase-contrast brightfield inverted Olympus microscope (CKX41, Olympus, Tokyo, Japan) fitted with a Panasonic camera (DMC-GH3) was used to capture images of the differentiating cells.

The number of neurite bearings per cell was quantified manually in 5–10 different fields of view and the results were expressed as a percentage.

### 2.11. Statistical analysis

Statistical data processing was conducted using the Statistical Package for the Social Sciences, SPSS 24.0 (SPSS, Chicago, IL, USA). Statistically significant differences (*p* < 0.05, *p* < 0.01) were calculated using a one-way ANOVA analysis followed by a post hoc Tukey's multiple comparison test. The independent variables in the study were the two different conditions of treatment.

## 3. Results

**Figure 1.**
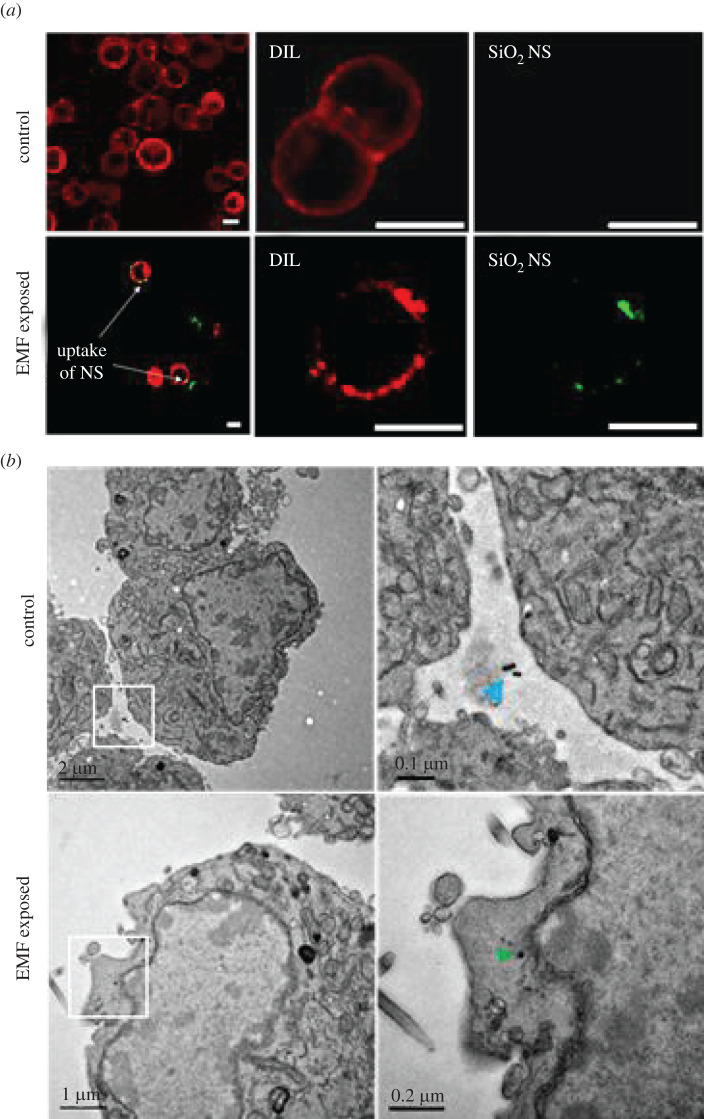
HF EMF-induced NS uptake by PC 12 cells was confirmed by CLSM and TEM. Green fluorescent FITC-silica NS (SiO_2_ NS) were used to assess membrane permeability in EMF-exposed and non-exposed (control) cells. The membrane phospholipids were stained using DIL (red). (*a*) Low magnification (left column) and high magnification (right columns) CLSM micrographs illustrating the uptake of the FITC-silica NS by EMF-exposed PC 12 cells. Green fluorescence confirms the presence of NS whereas the non-exposed control does not display internalization of the NS and no signal was detected from the FITC channel. NS uptake was confirmed from observation of approximately 50 cells (5–10 cells per 10 fields of view; 90% of the cell population exhibited uptake) [6]. (*b*) TEM images confirm the uptake of NS by the EMF-exposed cells. The blue arrow indicates Au NS external to the cell in control TEM images and green arrows indicate internalized Au NS in EMF-exposed cells.

PC 12 cell membrane permeability following exposure to EMFs of 18 GHz and internalization of NS was confirmed using both fluorescence and electron microscopy (figure 1). Our previous dosimetry work confirmed the PC 12 cells to have a specific absorption rate value of 1.17 kW kg^−1^, assuming no heat loss to the surrounding solution [6]. Some heat loss to the surrounding medium can be neglected as the constituents are similar to that of water with a specific heat of 4.18 kJ kg^−1^°C^−1^ [6]. In due course of the treatment, the temperature of the cell suspensions remained below 37°C. The average temperature of the cell suspension after one 30 s cycle of EMF exposure was recorded as 34.94 ± 1.73°C [6].

**Figure 2.**
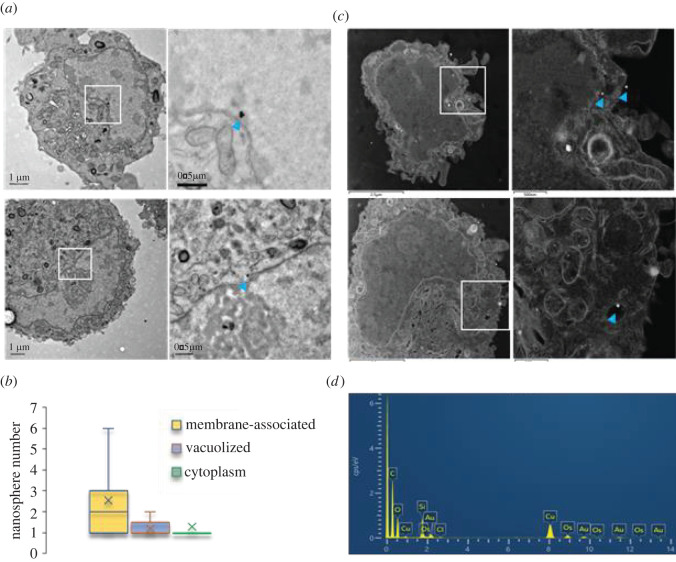
Localization of silica core–shell Au NS in PC 12 following HF EMF exposure of 18 GHz. (*a*) Low magnification (left panel) and high magnification (right panel) TEM micrographs of internalized Au NS. Au NS can be observed in the cytoplasm of PC 12 cells (blue arrows). (*b*) Quantification of the distribution of the relative numbers of Au NS isolated at the membrane, in vacuoles, or free within the cytoplasm as determined from analysis of TEM images of approximately 40 EMF-exposed cells. No internalization was detected in the non-exposed control group in comparison to the EMF-exposed cells (electronic supplementary material, figure S1). (*c*) Low magnification (left panel) and high magnification (right panel) STEM micrographs of Au NS localized in the cytoplasm, at the membrane, and within vacuoles in PC12 cells (indicated by blue arrows). (*d*) Representative EDS spectra confirming the presence of elemental Si and Au in the STEM micrographs in 2C, thus confirming the presence of Au NS.

CLSM micrographs in figure 1*a* confirm cell–NS interactions, although most NS appear to be membrane bound, suggesting membrane-associated uptake. Higher resolution imaging achieved by TEM (figure 1*b*) shows the NS to be inside the cell cytoplasm. By contrast, in the non-exposed control samples in both CLSM and TEM images, the NS are only located external to the cell membrane and are not membrane associated at all. Thus, EMF exposure may trigger increased cell–NS interaction, as evidenced in figure 1. After confirming the silica NS cell–particle interactions, the localization of the NS in the PC 12 cells was investigated further using both TEM and STEM-EDS analysis (figure 2).

TEM images in figure 2*a* show Au NS localized free within the cell cytoplasm. In both images, the NS appear close to the nuclear membrane. STEM-EDS micrographs further confirm the presence of the NS inside the PC 12 cells and the Au NS appear in vacuoles inside the cell cytoplasm (figure 2*c*). The corresponding elemental spectra obtained from EDS analysis exhibiting peaks for Si and Au further confirm that the EMF exposure of 18 GHz induced membrane permeability in the PC 12 cells whereas the non-exposed PC 12 cells did not exhibit NS internalization (electronic supplementary material, figure S2); the Au NS are external to the cell membrane, or not associated with the cells at all.

Quantification of the localization of Au NS revealed that the majority of observed Au NS were located close to the membrane or were membrane associated (figure 2*b*). Additionally, membrane-associated Au NS were more likely to appear in large aggregates of Au NS, i.e. as clusters of three or more Au NS. By contrast, those Au NS that were observed free within the cell cytoplasm or localized within vacuoles were generally single Au NS.

**Figure 3.**
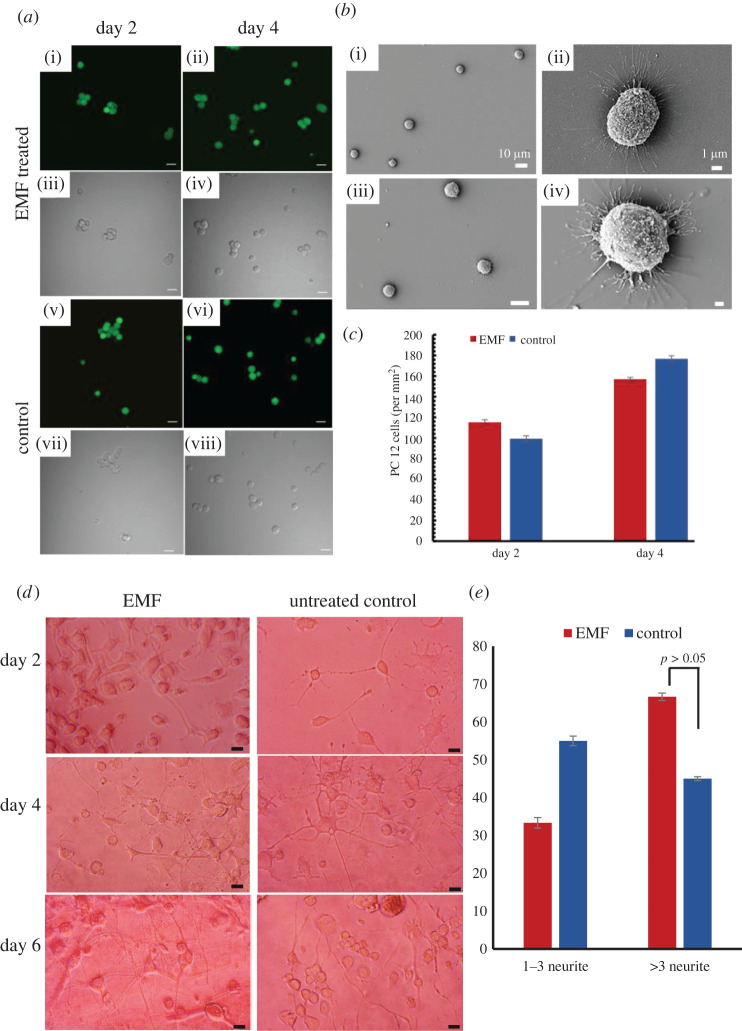
PC 12 cell viability following exposure to HF EMFs exposures of 18 GHz. (*a*) CLSM micrographs of PC 12 cells 2 and 4 days following EMF exposure and corresponding control (no EMF exposure). Cells were stained with SYBR green and ethidium bromide (Live/Dead) to assess cell health. Green cells are considered viable (i–ii, v–vi). Corresponding phase-contrast images (iii–iv, vii–viii) of the same cells show the preserved cell morphology. (*b*) SEM micrographs of the non-exposed control PC 12 cell sample and EMF-exposed cells show the preserved cell morphology. (*c*) Quantification of cell proliferation on day 2 and day 4 post-EMF exposure. There are no statistically significant differences in the EMF-exposed sample and the non-exposed control sample (*p* > 0.05). (*d*) Differentiation of PC 12 cells on days 2, 4 and 6 following exposure to 18 GHz EMF as visualized using phase-contrast microscopy. (*e*) Quantification of the number of neurite bearings per cell in the two experimental groups. No statistically significant difference was observed in the EMF-exposed and the non-exposed controls (*p* > 0.05).

We previously showed that PC 12 cells remained viable after transient membrane permeability [13]. In this work, we studied the viability of PC 12 cells after 2 and 4 days post-EMF exposure (the cell viability and morphology after day 1 and 5 are shown in electronic supplementary material, figure S2). It appeared that the PC 12 cells exhibited a healthy morphology (figure 3). The assessment of the PC 12 cells viability following exposure to EMFs (2–4 days) revealed no significant differences in cell viability and morphology in comparison to the non-exposed control group, as depicted in figure 3. The quantification of the cell numbers on day 2 and day 4 revealed no significant difference between the EMF-exposed and the control group (*p* = 0.34; *p* = 0.29, respectively), indicating that the cells proliferated normally post-EMF exposure.

Additionally, changes to PC 12 attachment and differentiation capability were assessed following exposure to 18 GHz EMF. No changes in PC 12 cell attachment and differentiation were observed following EMF exposure; approximately 90% of the cellular population exhibited differentiation following attachment onto collagen-coated surfaces in all the experimental samples, consistent with the non-treated control population. PC 12 cells in the presence of NGF concentrations of 50 ng mL^−1^ exhibited axonal outgrowth and the extent of neurite formation increased with time. As seen in figure 3*d*, day 6 displays the greatest extent of cellular differentiation, evidenced by significant neurite outgrowth. Upon analysis of the neurite outgrowth, more than 66.6 ± 1.0% of the EMF-treated cell population exhibited greater than three neurite bearings and 45 ± 0.5% of the control cell population exhibited greater than three neurite bearings. Although the EMF-exposed samples had (algebraically) more neurite bearings in comparison to the control sample, the results were not statistically significant (*p* = 0.75) (figure 3*e*).

## 4. Discussion

The results of this study confirmed that exposure of PC 12 cells to EMFs of 18 GHz induced increased membrane permeability without compromising the cells viability, and morphology [6], in agreement with our previous reports and similar to that observed in prokaryotic bacterial taxa [5,14].

Examples of other techniques that have been widely investigated to breach the cell membrane to deliver genetic material, drugs or other objects into the cell include electroporation, sonoporation, photoporation or mechanical stress. Electroporation causes an increase in transmembrane potential of the cell [15], irrespective of the cell type, leading to reversible pore formation that allows for DNA or other large molecules to enter the cell [13,15–19] by the application of external pulsed electric fields (PEFs) [20]. Sonoporation is a technique that increases cell membrane permeability by exposure to ultrasound leading to the formation of transient pores [21–24], whereas laser irradiation (photoporation) has also been demonstrated to efficiently introduce foreign DNA into cells in culture by way of increasing membrane permeability [25]. In this work, it seems that the membrane permeability induced through exposure to 18 GHz is due to an independent mechanism, and not related to those associated with membrane poration induced by electroporation, sonoporation, photoporation and mechanical stress [26–28].

Other routinely studied methods of cell permeabilization, in particular electroporation, suffer from significant drawbacks associated with a negative effect on normal cellular function and cell viability [19]. Additionally, PEFs are usually directed to cells in suspension, hence there is relatively little control over the area of electroporation [13]. By contrast, cell exposure of EMFs of 18 GHz leads to no observable detrimental effects with regard to cell morphology, attachment, proliferation and differentiation [6]. Another substantial advantage of the EMF-induced cell permeabilization technique is that the radiation is received equally by all cells in the monolayer which leads to uniform energy absorption by the entire population. In addition, the cell-protective effects from EMF have been investigated by various researchers [29–31]. Recent evidence has been provided that exposure to RF-EMF is capable of inducing beneficial effects *in vivo* and *in vitro* while protecting cells from damage arising from subsequent treatments with physical or chemical agents [29]. For example, studies have suggested that pre-exposure to non-ionizing EMF is able to induce a phenomenon similar to that adaptive response [30]. Thus, EMF exposure, in the range of 18 GHz, has potential to be used as a safe and effective method of cell permeabilization in the field of drug delivery and gene therapy.

However, the nature of membrane permeability induced by exposure to HF EMF remains unclear due to the lack of relevant studies. Few groups have studied the biological effects of long-term (6 h) exposures at low frequency (50 Hz) EMF using neuron-like cells [21–26]. Using Fourier transform infrared spectroscopic analysis, the authors found an increase in the stretching vibration of Amide I bands that were attributed to the α-helical component of proteins present in the cellular membrane [32]. The authors hypothesized that alignment of the α-helical component of membrane proteins in the phospholipid bilayer might induce an enlargement of the membrane channels and an increase in the flux of ions across the membrane as the result of long-term EMF (50 Hz) exposure [32]. It was also reported that the cell surface area increases following exposure to EMFs [33] and suggested that observed vibrational stretching of CH_2_ bands might contribute towards some morphological changes to the organization of the phospholipid bilayer and/or depolarization of the membrane. However, no direct comparison can be drawn between these studies and the current work due to the very different frequencies used.

## 5. Conclusion

In this work, the localization of Au NS in PC 12 cells following exposure to EMFs of 18 GHz was determined using a combination of fluorescence and electron microscopy. Au NS were only observed to be uptaken by PC 12 cells treated with HF EMFs of 18 GHz, in comparison to those PC 12 cells that did not undergo HF EMF exposure. Au NS were localized both free within the cytoplasm, enclosed within intracellular vesicles, and associated with the cell membrane. Therefore, the mechanism of cellular HF EMF-induced uptake of NS in the size range of 20–25 nm may be a combination of both active and passive translocation mechanisms. This work highlights that exposure to HF EMFs could be a promising alternative technique to facilitate a more effective delivery of drugs, genetic material and nanomaterials into cells.

## Data Availability

The data are available in the manuscript, in the electronic supplementary material data file and other supporting data have been uploaded as part of the electronic supplementary material [34].
